# Perchloratobis[1-(1,10-phenanthrolin-2-yl)-2-pyridone]zinc(II) perchlorate

**DOI:** 10.1107/S1600536809025355

**Published:** 2009-07-08

**Authors:** Qing Yun Liu, Qi Sheng Liu, Qing Ru Zhao

**Affiliations:** a School of Chemical & Environmental Engineering, Shandong University of Science and Technology, Qingdao 266510, People’s Republic of China; b Department of Chemistry, Shandong Normal University, Jinan 250014, People’s Republic of China

## Abstract

In the title mononuclear complex, [Zn(ClO_4_)(C_17_H_11_N_3_O)_2_]ClO_4_, the Zn^II^ ion is coordinated in a distorted octa­hedral geometry. The dihedral angles between the pyridine rings and the mean planes of the 1,10-phenanthroline ring system in each of the 1-(1,10-phenanthrolin-2-yl)-2-pyridone (PP) ligands is 24.51 (10)° for the tridendate PP ligand and 73.55 (6)° for the bidentate PP ligand. Within the mol­ecule there is a weak π–π inter­action between the pyridine ring of the bidentate ligand and the 1,10-phenanthroline ring system of the tridendate ligand with a centroid–centroid distance of 3.6383 (19) Å.

## Related literature

For a related crystal structure and background information, see: Liu *et al.* (2008 ▶).
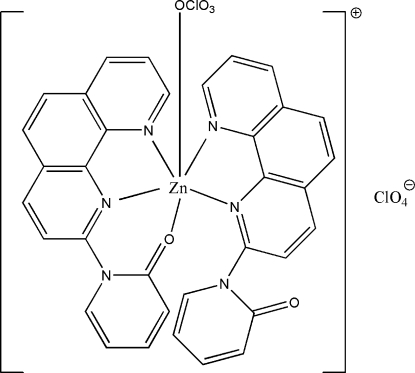


## Experimental

### 

#### Crystal data

[Zn(ClO_4_)(C_17_H_11_N_3_O)_2_]ClO_4_
*M* *_r_* = 810.85
Monoclinic, 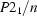

*a* = 12.998 (2) Å
*b* = 16.741 (3) Å
*c* = 14.680 (3) Å
β = 100.068 (2)°
*V* = 3145.2 (10) Å^3^
*Z* = 4
Mo *K*α radiation
μ = 1.03 mm^−1^
*T* = 298 K
0.45 × 0.38 × 0.26 mm

#### Data collection

Bruker SMART APEX CCD diffractometer
Absorption correction: multi-scan (*SADABS*; Sheldrick, 1996 ▶) *T*_min_ = 0.655, *T*_max_ = 0.776
16722 measured reflections
6163 independent reflections
5005 reflections with *I* > 2σ(*I*)
*R*_int_ = 0.028

#### Refinement

*R*[*F* ^2^ > 2σ(*F* ^2^)] = 0.044
*wR*(*F* ^2^) = 0.130
*S* = 1.08
6163 reflections
480 parameters
H-atom parameters constrained
Δρ_max_ = 0.82 e Å^−3^
Δρ_min_ = −0.63 e Å^−3^

### 

Data collection: *SMART* (Bruker, 1997 ▶); cell refinement: *SAINT* (Bruker, 1997 ▶); data reduction: *SAINT*; program(s) used to solve structure: *SHELXTL* (Sheldrick, 2008 ▶); program(s) used to refine structure: *SHELXTL*; molecular graphics: *SHELXTL*; software used to prepare material for publication: *SHELXTL*.

## Supplementary Material

Crystal structure: contains datablocks I, global. DOI: 10.1107/S1600536809025355/lh2853sup1.cif

Structure factors: contains datablocks I. DOI: 10.1107/S1600536809025355/lh2853Isup2.hkl

Additional supplementary materials:  crystallographic information; 3D view; checkCIF report

## Figures and Tables

**Fig. 1.**
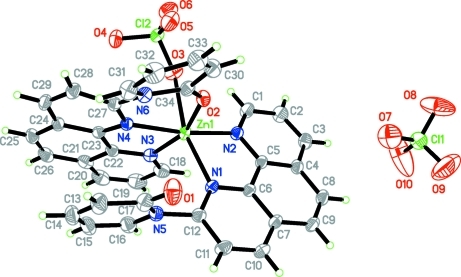
The asymmetric unit of (I) showing the atom numbering scheme with thermal ellipsoids drawn at the 30% probability level.

## supplementary crystallographic information

### Comment

Metal complexes containing derivatives of 1,10-phenanthroline as ligands play a pivotal role in the area of modern coordination chemistry. One of the first metal complexes containing the 1-(1,10-phenanthrolin-2-yl)-2-pyridone (PP) ligand was published recently (Liu *et al.*, 2008 and references cited within). Our interest in this area motivated us to synthesize the title complex, and here we report its crystal structure.

The asymmetric unit of the title compound (I) is shown in Fig. 1. The data of coordination bond lengths and associated angles (Table 1) indicate that the Zn^II^ ion is in a distorted octahedral geometry. The dihedral angles between pyridine rings and the mean planes of the 1,10-phenanthroline ring system in each of the 1-(1,10-phenanthrolin-2-yl)-2-pyridone (PP) ligands is 24.51 (10)° for the tridendate PP ligand and 73.55 (6)° for the bidentate PP ligand. There is a weak π–π interaction with *Cg*1···*Cg*2 = 3.6376 (19)Å and *Cg*1···*Cg*2_perp_ = 3.569 Å; α is 15.63° [*Cg*1 and *Cg*2 are the centroids of C23C24C27-C29/N4 ring and C13—C17/N5 ring, respectively; *Cg*1···*Cg*2^i^_perp_ is the perpendicular distance from ring *Cg*1 to ring *Cg*2; α is the dihedral angle between the *Cg*1 ring plane and the *Cg*2 ring plane].

### Experimental

Hydrated zinc perchlorate (0.2418 g, 0.65 mmol) and 1-(1,10-phenanthrolin-2-yl)-2-pyridone (0.1774 g, 0.65 mmol) were dissolved in 10 ml methanol, and the solution was stirred for a few minutes. Yellow single crystals were obtained after the filtrate had been allowed to stand at room temperature for one week.

### Refinement

All H atoms were placed in calculated positions, and refined as riding, with C—H = 0.93 Å and *U*_iso_(H) = 1.2_eq_(C).

### Crystal data

**Table:**

[Zn(ClO_4_)(C_17_H_11_N_3_O)_2_]ClO_4_	*F*(000) = 1648
*M**_r_* = 810.85	*D*_x_ = 1.712 Mg m^−^^3^
Monoclinic, *P*2_1_/*n*	Mo *K*α radiation, λ = 0.71073 Å
Hall symbol: -P 2yn	Cell parameters from 6273 reflections
*a* = 12.998 (2) Å	θ = 2.3–27.0°
*b* = 16.741 (3) Å	µ = 1.02 mm^−^^1^
*c* = 14.680 (3) Å	*T* = 298 K
β = 100.068 (2)°	Block, yellow
*V* = 3145.2 (10) Å^3^	0.45 × 0.38 × 0.26 mm
*Z* = 4	

### Data collection

**Table:**

Bruker SMART APEX CCD diffractometer	6163 independent reflections
Radiation source: fine-focus sealed tube	5005 reflections with *I* > 2σ(*I*)
graphite	*R*_int_ = 0.028
φ and ω scans	θ_max_ = 26.0°, θ_min_ = 1.9°
Absorption correction: multi-scan (*SADABS*; Sheldrick, 1996)	*h* = −12→16
*T*_min_ = 0.655, *T*_max_ = 0.776	*k* = −20→19
16722 measured reflections	*l* = −17→18

### Refinement

**Table:**

Refinement on *F*^2^	Primary atom site location: structure-invariant direct methods
Least-squares matrix: full	Secondary atom site location: difference Fourier map
*R*[*F*^2^ > 2σ(*F*^2^)] = 0.044	Hydrogen site location: inferred from neighbouring sites
*wR*(*F*^2^) = 0.130	H-atom parameters constrained
*S* = 1.08	*w* = 1/[σ^2^(*F*_o_^2^) + (0.0839*P*)^2^ + 0.2024*P*] where *P* = (*F*_o_^2^ + 2*F*_c_^2^)/3
6163 reflections	(Δ/σ)_max_ = 0.001
480 parameters	Δρ_max_ = 0.82 e Å^−^^3^
0 restraints	Δρ_min_ = −0.62 e Å^−^^3^

### Special details

**Table:**

Geometry. All e.s.d.'s (except the e.s.d. in the dihedral angle between two l.s. planes) are estimated using the full covariance matrix. The cell e.s.d.'s are taken into account individually in the estimation of e.s.d.'s in distances, angles and torsion angles; correlations between e.s.d.'s in cell parameters are only used when they are defined by crystal symmetry. An approximate (isotropic) treatment of cell e.s.d.'s is used for estimating e.s.d.'s involving l.s. planes.
Refinement. Refinement of *F*^2^ against ALL reflections. The weighted *R*-factor *wR* and goodness of fit *S* are based on *F*^2^, conventional *R*-factors *R* are based on *F*, with *F* set to zero for negative *F*^2^. The threshold expression of *F*^2^ > σ(*F*^2^) is used only for calculating *R*-factors(gt) *etc*. and is not relevant to the choice of reflections for refinement. *R*-factors based on *F*^2^ are statistically about twice as large as those based on *F*, and *R*- factors based on ALL data will be even larger.

### Fractional atomic coordinates and isotropic or equivalent isotropic displacement parameters (Å^2^)

**Table:**

	*x*	*y*	*z*	*U*_iso_*/*U*_eq_	
C1	0.1039 (2)	0.10171 (18)	0.5679 (2)	0.0474 (7)	
H1	0.0681	0.0630	0.5955	0.057*	
C2	0.1066 (3)	0.09526 (19)	0.4745 (2)	0.0535 (8)	
H2	0.0742	0.0524	0.4408	0.064*	
C3	0.1568 (2)	0.1520 (2)	0.4320 (2)	0.0505 (8)	
H3	0.1573	0.1490	0.3688	0.061*	
C4	0.2076 (2)	0.21493 (17)	0.48458 (19)	0.0395 (6)	
C5	0.20322 (19)	0.21620 (15)	0.57950 (18)	0.0331 (6)	
C6	0.25810 (19)	0.27716 (15)	0.63721 (18)	0.0313 (5)	
C7	0.3130 (2)	0.33617 (16)	0.5965 (2)	0.0370 (6)	
C8	0.2632 (2)	0.27598 (19)	0.4458 (2)	0.0450 (7)	
H8	0.2645	0.2759	0.3827	0.054*	
C9	0.3140 (2)	0.33375 (18)	0.4999 (2)	0.0439 (7)	
H9	0.3505	0.3728	0.4735	0.053*	
C10	0.3663 (2)	0.39396 (16)	0.6561 (2)	0.0440 (7)	
H10	0.4015	0.4351	0.6320	0.053*	
C11	0.3670 (2)	0.39036 (18)	0.7479 (2)	0.0481 (8)	
H11	0.4024	0.4286	0.7873	0.058*	
C12	0.3138 (2)	0.32829 (16)	0.7828 (2)	0.0382 (6)	
C13	0.2868 (3)	0.3608 (2)	1.0268 (2)	0.0610 (9)	
H13	0.2537	0.3943	1.0632	0.073*	
C14	0.3496 (3)	0.3029 (2)	1.0683 (2)	0.0624 (9)	
H14	0.3589	0.2969	1.1322	0.075*	
C15	0.4010 (3)	0.25162 (19)	1.0158 (2)	0.0590 (9)	
H15	0.4448	0.2117	1.0446	0.071*	
C16	0.3864 (2)	0.26046 (18)	0.9239 (2)	0.0498 (7)	
H16	0.4205	0.2264	0.8888	0.060*	
C17	0.2688 (3)	0.37307 (19)	0.9287 (2)	0.0507 (8)	
C18	0.3225 (2)	0.04946 (19)	0.7636 (2)	0.0497 (8)	
H18	0.3201	0.0605	0.7011	0.060*	
C19	0.3877 (2)	−0.0110 (2)	0.8049 (3)	0.0617 (9)	
H19	0.4280	−0.0401	0.7702	0.074*	
C20	0.3924 (3)	−0.02735 (19)	0.8950 (3)	0.0579 (9)	
H20	0.4367	−0.0675	0.9225	0.069*	
C21	0.3313 (2)	0.01527 (16)	0.9483 (2)	0.0447 (7)	
C22	0.2667 (2)	0.07564 (15)	0.90174 (19)	0.0349 (6)	
C23	0.2012 (2)	0.12133 (15)	0.95101 (18)	0.0330 (6)	
C24	0.1966 (2)	0.10129 (16)	1.04237 (19)	0.0381 (6)	
C25	0.2612 (2)	0.03954 (18)	1.0871 (2)	0.0484 (7)	
H25	0.2578	0.0266	1.1481	0.058*	
C26	0.3269 (2)	−0.00048 (18)	1.0429 (2)	0.0525 (8)	
H26	0.3704	−0.0392	1.0745	0.063*	
C27	0.0828 (2)	0.22394 (15)	0.94907 (18)	0.0334 (6)	
C28	0.0674 (2)	0.20372 (18)	1.0379 (2)	0.0433 (7)	
H28	0.0177	0.2306	1.0648	0.052*	
C29	0.1256 (2)	0.14452 (17)	1.0849 (2)	0.0437 (7)	
H29	0.1183	0.1327	1.1454	0.052*	
C30	−0.0805 (3)	0.35800 (18)	0.7785 (2)	0.0520 (8)	
H6	−0.1105	0.3600	0.7162	0.062*	
C31	−0.0005 (3)	0.35034 (18)	0.9622 (2)	0.0501 (8)	
H31	0.0285	0.3494	1.0248	0.060*	
C32	−0.0680 (3)	0.40880 (19)	0.9300 (3)	0.0584 (9)	
H32	−0.0858	0.4473	0.9701	0.070*	
C33	−0.1111 (3)	0.41155 (19)	0.8361 (3)	0.0588 (9)	
H33	−0.1607	0.4501	0.8137	0.071*	
C34	−0.0037 (2)	0.29806 (15)	0.8084 (2)	0.0378 (6)	
Cl1	0.05475 (7)	0.33552 (5)	0.23847 (6)	0.0555 (2)	
Cl2	−0.06238 (5)	0.05947 (4)	0.78950 (5)	0.03671 (17)	
N1	0.25867 (16)	0.27400 (12)	0.72957 (15)	0.0331 (5)	
N2	0.15024 (18)	0.16100 (13)	0.62039 (15)	0.0359 (5)	
N3	0.26358 (17)	0.09182 (13)	0.81097 (16)	0.0371 (5)	
N4	0.14672 (16)	0.18319 (11)	0.90497 (15)	0.0306 (5)	
N5	0.32214 (18)	0.31891 (13)	0.88079 (16)	0.0398 (5)	
N6	0.02718 (18)	0.29118 (13)	0.90430 (16)	0.0378 (5)	
O1	0.2131 (2)	0.42378 (16)	0.88607 (18)	0.0763 (8)	
O2	0.03168 (15)	0.25565 (11)	0.75179 (13)	0.0405 (4)	
O3	0.03026 (16)	0.07488 (12)	0.75009 (15)	0.0481 (5)	
O4	−0.03149 (17)	0.03559 (13)	0.88350 (15)	0.0530 (5)	
O5	−0.12309 (19)	0.12974 (14)	0.78672 (19)	0.0675 (7)	
O6	−0.1177 (2)	−0.00388 (17)	0.73801 (17)	0.0728 (7)	
O7	0.0442 (3)	0.3557 (2)	0.3300 (2)	0.1018 (10)	
O8	−0.0400 (3)	0.3197 (3)	0.1845 (3)	0.147 (2)	
O9	0.0971 (5)	0.3960 (3)	0.1999 (4)	0.204 (3)	
O10	0.1218 (5)	0.2717 (3)	0.2398 (3)	0.208 (3)	
Zn1	0.15585 (2)	0.180720 (18)	0.76169 (2)	0.03378 (13)	

### Atomic displacement parameters (Å^2^)

**Table:**

	*U*^11^	*U*^22^	*U*^33^	*U*^12^	*U*^13^	*U*^23^
C1	0.0607 (19)	0.0447 (17)	0.0382 (17)	−0.0144 (14)	0.0122 (14)	−0.0046 (13)
C2	0.069 (2)	0.0489 (18)	0.0416 (18)	−0.0119 (15)	0.0075 (15)	−0.0141 (14)
C3	0.0567 (19)	0.064 (2)	0.0315 (16)	−0.0010 (16)	0.0106 (14)	−0.0058 (14)
C4	0.0419 (15)	0.0451 (15)	0.0323 (15)	0.0039 (12)	0.0089 (12)	0.0034 (12)
C5	0.0349 (14)	0.0356 (14)	0.0302 (14)	0.0023 (11)	0.0093 (11)	0.0015 (11)
C6	0.0317 (13)	0.0307 (13)	0.0333 (14)	0.0025 (10)	0.0102 (10)	0.0032 (10)
C7	0.0364 (14)	0.0340 (13)	0.0433 (16)	0.0027 (11)	0.0140 (12)	0.0085 (12)
C8	0.0476 (16)	0.0552 (18)	0.0350 (16)	0.0061 (14)	0.0150 (13)	0.0072 (13)
C9	0.0440 (16)	0.0451 (16)	0.0465 (18)	0.0024 (13)	0.0188 (13)	0.0144 (13)
C10	0.0473 (17)	0.0347 (14)	0.0526 (19)	−0.0091 (12)	0.0162 (14)	0.0057 (13)
C11	0.0525 (18)	0.0362 (15)	0.057 (2)	−0.0146 (13)	0.0129 (15)	−0.0034 (13)
C12	0.0393 (15)	0.0383 (15)	0.0369 (15)	−0.0046 (11)	0.0069 (12)	−0.0014 (11)
C13	0.081 (2)	0.063 (2)	0.0414 (19)	−0.0055 (19)	0.0151 (17)	−0.0133 (16)
C14	0.081 (3)	0.066 (2)	0.0362 (18)	−0.0190 (19)	−0.0007 (17)	0.0011 (16)
C15	0.065 (2)	0.054 (2)	0.051 (2)	−0.0065 (17)	−0.0107 (16)	0.0078 (16)
C16	0.0538 (18)	0.0426 (16)	0.051 (2)	−0.0052 (14)	0.0020 (15)	−0.0002 (14)
C17	0.061 (2)	0.0456 (17)	0.0462 (19)	−0.0025 (15)	0.0113 (15)	−0.0036 (14)
C18	0.0421 (16)	0.0528 (18)	0.055 (2)	0.0031 (14)	0.0107 (14)	−0.0121 (15)
C19	0.0441 (18)	0.058 (2)	0.084 (3)	0.0136 (15)	0.0133 (17)	−0.0142 (19)
C20	0.0464 (18)	0.0453 (18)	0.077 (3)	0.0107 (14)	−0.0031 (16)	−0.0034 (17)
C21	0.0391 (15)	0.0338 (15)	0.057 (2)	−0.0004 (12)	−0.0026 (13)	0.0009 (13)
C22	0.0316 (13)	0.0317 (13)	0.0396 (16)	−0.0043 (11)	0.0006 (11)	0.0016 (11)
C23	0.0340 (13)	0.0300 (13)	0.0330 (14)	−0.0089 (10)	0.0007 (11)	0.0028 (11)
C24	0.0436 (16)	0.0362 (14)	0.0327 (15)	−0.0108 (12)	0.0022 (12)	0.0042 (11)
C25	0.0531 (18)	0.0465 (17)	0.0410 (17)	−0.0109 (14)	−0.0047 (14)	0.0134 (13)
C26	0.0464 (18)	0.0407 (16)	0.063 (2)	−0.0018 (14)	−0.0124 (15)	0.0181 (15)
C27	0.0384 (14)	0.0308 (13)	0.0309 (14)	−0.0059 (11)	0.0053 (11)	−0.0012 (11)
C28	0.0512 (17)	0.0440 (16)	0.0374 (16)	−0.0071 (13)	0.0153 (13)	−0.0065 (13)
C29	0.0548 (18)	0.0459 (17)	0.0314 (15)	−0.0124 (14)	0.0096 (13)	0.0029 (12)
C30	0.0535 (19)	0.0446 (17)	0.057 (2)	0.0101 (14)	0.0080 (15)	0.0073 (15)
C31	0.062 (2)	0.0409 (16)	0.0491 (19)	0.0017 (14)	0.0137 (15)	−0.0110 (14)
C32	0.068 (2)	0.0382 (17)	0.073 (3)	0.0093 (15)	0.0222 (18)	−0.0109 (16)
C33	0.062 (2)	0.0423 (17)	0.075 (3)	0.0170 (15)	0.0190 (18)	0.0094 (16)
C34	0.0427 (15)	0.0299 (13)	0.0417 (16)	−0.0015 (11)	0.0101 (12)	0.0042 (12)
Cl1	0.0624 (5)	0.0548 (5)	0.0495 (5)	−0.0031 (4)	0.0100 (4)	−0.0099 (4)
Cl2	0.0390 (4)	0.0355 (3)	0.0378 (4)	−0.0005 (3)	0.0129 (3)	0.0017 (3)
N1	0.0370 (12)	0.0304 (11)	0.0330 (12)	−0.0025 (9)	0.0092 (9)	0.0011 (9)
N2	0.0419 (13)	0.0348 (11)	0.0317 (12)	−0.0072 (10)	0.0082 (10)	−0.0019 (9)
N3	0.0361 (12)	0.0350 (12)	0.0403 (14)	−0.0001 (10)	0.0070 (10)	−0.0024 (10)
N4	0.0330 (11)	0.0280 (11)	0.0309 (12)	−0.0038 (8)	0.0054 (9)	−0.0003 (8)
N5	0.0455 (14)	0.0369 (12)	0.0362 (13)	−0.0077 (10)	0.0046 (10)	−0.0012 (10)
N6	0.0424 (13)	0.0329 (12)	0.0394 (14)	0.0003 (10)	0.0105 (10)	−0.0020 (10)
O1	0.098 (2)	0.0762 (17)	0.0574 (16)	0.0310 (16)	0.0201 (14)	0.0083 (14)
O2	0.0461 (11)	0.0398 (11)	0.0353 (11)	0.0070 (8)	0.0063 (9)	0.0018 (8)
O3	0.0490 (12)	0.0427 (11)	0.0591 (14)	−0.0064 (9)	0.0277 (10)	−0.0052 (10)
O4	0.0644 (14)	0.0554 (13)	0.0409 (12)	0.0072 (11)	0.0135 (10)	0.0092 (10)
O5	0.0689 (15)	0.0610 (15)	0.0807 (18)	0.0316 (12)	0.0357 (13)	0.0232 (13)
O6	0.0808 (17)	0.0791 (17)	0.0607 (16)	−0.0412 (15)	0.0188 (13)	−0.0191 (13)
O7	0.119 (3)	0.123 (3)	0.063 (2)	−0.016 (2)	0.0161 (17)	−0.0431 (18)
O8	0.067 (2)	0.260 (5)	0.115 (3)	−0.034 (3)	0.021 (2)	−0.117 (3)
O9	0.217 (5)	0.220 (5)	0.181 (5)	−0.126 (5)	0.050 (4)	0.049 (4)
O10	0.331 (7)	0.161 (4)	0.094 (3)	0.145 (5)	−0.069 (4)	−0.068 (3)
Zn1	0.0430 (2)	0.03160 (19)	0.02842 (19)	−0.00084 (12)	0.01094 (14)	0.00178 (12)

### Geometric parameters (Å, °)

**Table:**

C1—N2	1.334 (4)	C21—C22	1.412 (4)
C1—C2	1.382 (4)	C21—C26	1.424 (5)
C1—H1	0.9300	C22—N3	1.354 (3)
C2—C3	1.365 (4)	C22—C23	1.431 (4)
C2—H2	0.9300	C23—N4	1.365 (3)
C3—C4	1.401 (4)	C23—C24	1.394 (4)
C3—H3	0.9300	C24—C29	1.402 (4)
C4—C5	1.405 (4)	C24—C25	1.418 (4)
C4—C8	1.427 (4)	C25—C26	1.339 (5)
C5—N2	1.354 (3)	C25—H25	0.9300
C5—C6	1.434 (4)	C26—H26	0.9300
C6—N1	1.356 (3)	C27—N4	1.328 (3)
C6—C7	1.411 (3)	C27—C28	1.396 (4)
C7—C10	1.403 (4)	C27—N6	1.434 (3)
C7—C9	1.421 (4)	C28—C29	1.359 (4)
C8—C9	1.349 (4)	C28—H28	0.9300
C8—H8	0.9300	C29—H29	0.9300
C9—H9	0.9300	C30—C33	1.340 (5)
C10—C11	1.348 (4)	C30—C34	1.429 (4)
C10—H10	0.9300	C30—H6	0.9300
C11—C12	1.395 (4)	C31—C32	1.344 (5)
C11—H11	0.9300	C31—N6	1.393 (4)
C12—N1	1.324 (3)	C31—H31	0.9300
C12—N5	1.432 (4)	C32—C33	1.395 (5)
C13—C14	1.342 (5)	C32—H32	0.9300
C13—C17	1.433 (5)	C33—H33	0.9300
C13—H13	0.9300	C34—O2	1.241 (3)
C14—C15	1.398 (5)	C34—N6	1.400 (4)
C14—H14	0.9300	Cl1—O9	1.326 (4)
C15—C16	1.338 (4)	Cl1—O8	1.369 (3)
C15—H15	0.9300	Cl1—O10	1.377 (4)
C16—N5	1.368 (4)	Cl1—O7	1.416 (3)
C16—H16	0.9300	Cl2—O5	1.413 (2)
C17—O1	1.216 (4)	Cl2—O6	1.423 (2)
C17—N5	1.403 (4)	Cl2—O4	1.425 (2)
C18—N3	1.327 (4)	Cl2—O3	1.4473 (19)
C18—C19	1.389 (5)	N1—Zn1	2.160 (2)
C18—H18	0.9300	N2—Zn1	2.089 (2)
C19—C20	1.341 (5)	N3—Zn1	2.084 (2)
C19—H19	0.9300	N4—Zn1	2.128 (2)
C20—C21	1.403 (4)	O2—Zn1	2.0291 (19)
C20—H20	0.9300	O3—Zn1	2.395 (2)
			
N2—C1—C2	122.9 (3)	C25—C26—C21	121.2 (3)
N2—C1—H1	118.5	C25—C26—H26	119.4
C2—C1—H1	118.5	C21—C26—H26	119.4
C3—C2—C1	119.8 (3)	N4—C27—C28	122.4 (2)
C3—C2—H2	120.1	N4—C27—N6	119.2 (2)
C1—C2—H2	120.1	C28—C27—N6	118.4 (2)
C2—C3—C4	119.2 (3)	C29—C28—C27	119.5 (3)
C2—C3—H3	120.4	C29—C28—H28	120.2
C4—C3—H3	120.4	C27—C28—H28	120.2
C3—C4—C5	117.5 (3)	C28—C29—C24	120.1 (3)
C3—C4—C8	122.8 (3)	C28—C29—H29	120.0
C5—C4—C8	119.7 (3)	C24—C29—H29	120.0
N2—C5—C4	122.8 (2)	C33—C30—C34	123.0 (3)
N2—C5—C6	117.6 (2)	C33—C30—H6	118.5
C4—C5—C6	119.6 (2)	C34—C30—H6	118.5
N1—C6—C7	122.4 (2)	C32—C31—N6	121.7 (3)
N1—C6—C5	118.6 (2)	C32—C31—H31	119.2
C7—C6—C5	119.0 (2)	N6—C31—H31	119.2
C10—C7—C6	116.7 (3)	C31—C32—C33	119.8 (3)
C10—C7—C9	123.5 (2)	C31—C32—H32	120.1
C6—C7—C9	119.8 (3)	C33—C32—H32	120.1
C9—C8—C4	120.6 (3)	C30—C33—C32	119.3 (3)
C9—C8—H8	119.7	C30—C33—H33	120.4
C4—C8—H8	119.7	C32—C33—H33	120.4
C8—C9—C7	121.3 (3)	O2—C34—N6	123.5 (2)
C8—C9—H9	119.4	O2—C34—C30	121.2 (3)
C7—C9—H9	119.4	N6—C34—C30	115.4 (3)
C11—C10—C7	120.6 (3)	O9—Cl1—O8	107.5 (4)
C11—C10—H10	119.7	O9—Cl1—O10	106.8 (4)
C7—C10—H10	119.7	O8—Cl1—O10	111.2 (3)
C10—C11—C12	119.0 (3)	O9—Cl1—O7	109.8 (3)
C10—C11—H11	120.5	O8—Cl1—O7	111.6 (2)
C12—C11—H11	120.5	O10—Cl1—O7	109.8 (2)
N1—C12—C11	123.1 (3)	O5—Cl2—O6	112.17 (18)
N1—C12—N5	117.1 (2)	O5—Cl2—O4	108.84 (14)
C11—C12—N5	119.7 (3)	O6—Cl2—O4	109.63 (15)
C14—C13—C17	122.6 (3)	O5—Cl2—O3	109.85 (13)
C14—C13—H13	118.7	O6—Cl2—O3	107.41 (14)
C17—C13—H13	118.7	O4—Cl2—O3	108.88 (13)
C13—C14—C15	120.3 (3)	C12—N1—C6	118.1 (2)
C13—C14—H14	119.8	C12—N1—Zn1	131.17 (18)
C15—C14—H14	119.8	C6—N1—Zn1	110.56 (16)
C16—C15—C14	119.4 (3)	C1—N2—C5	117.8 (2)
C16—C15—H15	120.3	C1—N2—Zn1	128.65 (19)
C14—C15—H15	120.3	C5—N2—Zn1	113.59 (17)
C15—C16—N5	120.8 (3)	C18—N3—C22	119.3 (3)
C15—C16—H16	119.6	C18—N3—Zn1	127.9 (2)
N5—C16—H16	119.6	C22—N3—Zn1	112.77 (17)
O1—C17—N5	119.7 (3)	C27—N4—C23	117.6 (2)
O1—C17—C13	126.6 (3)	C27—N4—Zn1	129.78 (18)
N5—C17—C13	113.8 (3)	C23—N4—Zn1	111.17 (16)
N3—C18—C19	121.6 (3)	C16—N5—C17	123.1 (3)
N3—C18—H18	119.2	C16—N5—C12	118.4 (2)
C19—C18—H18	119.2	C17—N5—C12	118.4 (2)
C20—C19—C18	119.9 (3)	C31—N6—C34	119.7 (2)
C20—C19—H19	120.0	C31—N6—C27	116.2 (2)
C18—C19—H19	120.0	C34—N6—C27	124.0 (2)
C19—C20—C21	120.9 (3)	C34—O2—Zn1	133.58 (19)
C19—C20—H20	119.6	Cl2—O3—Zn1	135.05 (12)
C21—C20—H20	119.6	O2—Zn1—N3	160.72 (8)
C20—C21—C22	116.2 (3)	O2—Zn1—N2	97.89 (8)
C20—C21—C26	124.9 (3)	N3—Zn1—N2	97.84 (9)
C22—C21—C26	118.9 (3)	O2—Zn1—N4	82.97 (8)
N3—C22—C21	122.2 (3)	N3—Zn1—N4	79.69 (8)
N3—C22—C23	118.2 (2)	N2—Zn1—N4	170.52 (8)
C21—C22—C23	119.6 (3)	O2—Zn1—N1	92.99 (8)
N4—C23—C24	123.6 (2)	N3—Zn1—N1	100.85 (8)
N4—C23—C22	117.3 (2)	N2—Zn1—N1	79.22 (8)
C24—C23—C22	119.1 (2)	N4—Zn1—N1	110.20 (8)
C23—C24—C29	116.5 (3)	O2—Zn1—O3	85.91 (8)
C23—C24—C25	120.0 (3)	N3—Zn1—O3	84.52 (8)
C29—C24—C25	123.5 (3)	N2—Zn1—O3	84.69 (8)
C26—C25—C24	121.1 (3)	N4—Zn1—O3	85.96 (7)
C26—C25—H25	119.4	N1—Zn1—O3	163.57 (8)
C24—C25—H25	119.4		
			
N2—C1—C2—C3	−1.2 (5)	N6—C27—N4—C23	177.7 (2)
C1—C2—C3—C4	1.9 (5)	C28—C27—N4—Zn1	162.4 (2)
C2—C3—C4—C5	−0.4 (4)	N6—C27—N4—Zn1	−17.5 (3)
C2—C3—C4—C8	179.0 (3)	C24—C23—N4—C27	−2.9 (4)
C3—C4—C5—N2	−1.8 (4)	C22—C23—N4—C27	177.5 (2)
C8—C4—C5—N2	178.8 (3)	C24—C23—N4—Zn1	−170.4 (2)
C3—C4—C5—C6	177.1 (3)	C22—C23—N4—Zn1	10.1 (3)
C8—C4—C5—C6	−2.4 (4)	C15—C16—N5—C17	0.6 (4)
N2—C5—C6—N1	3.4 (4)	C15—C16—N5—C12	176.8 (3)
C4—C5—C6—N1	−175.5 (2)	O1—C17—N5—C16	179.7 (3)
N2—C5—C6—C7	−179.2 (2)	C13—C17—N5—C16	−0.8 (4)
C4—C5—C6—C7	1.9 (4)	O1—C17—N5—C12	3.6 (4)
N1—C6—C7—C10	−2.1 (4)	C13—C17—N5—C12	−177.0 (3)
C5—C6—C7—C10	−179.3 (2)	N1—C12—N5—C16	74.3 (3)
N1—C6—C7—C9	176.6 (2)	C11—C12—N5—C16	−102.5 (3)
C5—C6—C7—C9	−0.7 (4)	N1—C12—N5—C17	−109.3 (3)
C3—C4—C8—C9	−177.6 (3)	C11—C12—N5—C17	73.9 (4)
C5—C4—C8—C9	1.8 (4)	C32—C31—N6—C34	9.1 (4)
C4—C8—C9—C7	−0.6 (4)	C32—C31—N6—C27	−168.1 (3)
C10—C7—C9—C8	178.6 (3)	O2—C34—N6—C31	167.6 (3)
C6—C7—C9—C8	0.1 (4)	C30—C34—N6—C31	−12.5 (4)
C6—C7—C10—C11	2.3 (4)	O2—C34—N6—C27	−15.4 (4)
C9—C7—C10—C11	−176.3 (3)	C30—C34—N6—C27	164.4 (2)
C7—C10—C11—C12	−0.1 (5)	N4—C27—N6—C31	−153.0 (2)
C10—C11—C12—N1	−2.7 (5)	C28—C27—N6—C31	27.1 (4)
C10—C11—C12—N5	173.8 (3)	N4—C27—N6—C34	30.0 (4)
C17—C13—C14—C15	0.2 (5)	C28—C27—N6—C34	−150.0 (3)
C13—C14—C15—C16	−0.5 (5)	N6—C34—O2—Zn1	−13.4 (4)
C14—C15—C16—N5	0.0 (5)	C30—C34—O2—Zn1	166.7 (2)
C14—C13—C17—O1	179.8 (4)	O5—Cl2—O3—Zn1	43.3 (2)
C14—C13—C17—N5	0.4 (5)	O6—Cl2—O3—Zn1	165.51 (19)
N3—C18—C19—C20	−0.3 (5)	O4—Cl2—O3—Zn1	−75.8 (2)
C18—C19—C20—C21	0.7 (5)	C34—O2—Zn1—N3	45.0 (4)
C19—C20—C21—C22	−0.5 (5)	C34—O2—Zn1—N2	−170.5 (2)
C19—C20—C21—C26	176.6 (3)	C34—O2—Zn1—N4	19.0 (2)
C20—C21—C22—N3	−0.1 (4)	C34—O2—Zn1—N1	−91.0 (2)
C26—C21—C22—N3	−177.4 (2)	C34—O2—Zn1—O3	105.4 (2)
C20—C21—C22—C23	179.4 (2)	C18—N3—Zn1—O2	156.0 (3)
C26—C21—C22—C23	2.1 (4)	C22—N3—Zn1—O2	−20.7 (4)
N3—C22—C23—N4	−5.7 (3)	C18—N3—Zn1—N2	11.6 (2)
C21—C22—C23—N4	174.8 (2)	C22—N3—Zn1—N2	−165.17 (17)
N3—C22—C23—C24	174.7 (2)	C18—N3—Zn1—N4	−177.7 (2)
C21—C22—C23—C24	−4.8 (4)	C22—N3—Zn1—N4	5.57 (17)
N4—C23—C24—C29	4.8 (4)	C18—N3—Zn1—N1	−68.9 (2)
C22—C23—C24—C29	−175.7 (2)	C22—N3—Zn1—N1	114.38 (18)
N4—C23—C24—C25	−175.7 (2)	C18—N3—Zn1—O3	95.4 (2)
C22—C23—C24—C25	3.9 (4)	C22—N3—Zn1—O3	−81.31 (18)
C23—C24—C25—C26	−0.2 (4)	C1—N2—Zn1—O2	−94.4 (3)
C29—C24—C25—C26	179.3 (3)	C5—N2—Zn1—O2	86.92 (19)
C24—C25—C26—C21	−2.6 (5)	C1—N2—Zn1—N3	74.4 (3)
C20—C21—C26—C25	−175.4 (3)	C5—N2—Zn1—N3	−104.27 (19)
C22—C21—C26—C25	1.6 (4)	C1—N2—Zn1—N4	0.2 (7)
N4—C27—C28—C29	5.6 (4)	C5—N2—Zn1—N4	−178.5 (4)
N6—C27—C28—C29	−174.5 (3)	C1—N2—Zn1—N1	174.0 (3)
C27—C28—C29—C24	−3.4 (4)	C5—N2—Zn1—N1	−4.64 (18)
C23—C24—C29—C28	−1.5 (4)	C1—N2—Zn1—O3	−9.3 (3)
C25—C24—C29—C28	179.0 (3)	C5—N2—Zn1—O3	172.02 (19)
N6—C31—C32—C33	−0.7 (5)	C27—N4—Zn1—O2	−2.4 (2)
C34—C30—C33—C32	−0.9 (5)	C23—N4—Zn1—O2	163.13 (17)
C31—C32—C33—C30	−3.4 (5)	C27—N4—Zn1—N3	−173.9 (2)
C33—C30—C34—O2	−171.4 (3)	C23—N4—Zn1—N3	−8.39 (16)
C33—C30—C34—N6	8.8 (4)	C27—N4—Zn1—N2	−98.2 (5)
C11—C12—N1—C6	3.0 (4)	C23—N4—Zn1—N2	67.3 (6)
N5—C12—N1—C6	−173.7 (2)	C27—N4—Zn1—N1	88.2 (2)
C11—C12—N1—Zn1	−172.3 (2)	C23—N4—Zn1—N1	−106.26 (16)
N5—C12—N1—Zn1	11.1 (4)	C27—N4—Zn1—O3	−88.7 (2)
C7—C6—N1—C12	−0.5 (4)	C23—N4—Zn1—O3	76.77 (16)
C5—C6—N1—C12	176.8 (2)	C12—N1—Zn1—O2	84.3 (2)
C7—C6—N1—Zn1	175.66 (19)	C6—N1—Zn1—O2	−91.27 (17)
C5—C6—N1—Zn1	−7.0 (3)	C12—N1—Zn1—N3	−82.2 (2)
C2—C1—N2—C5	−0.9 (4)	C6—N1—Zn1—N3	102.23 (17)
C2—C1—N2—Zn1	−179.6 (2)	C12—N1—Zn1—N2	−178.3 (3)
C4—C5—N2—C1	2.5 (4)	C6—N1—Zn1—N2	6.21 (16)
C6—C5—N2—C1	−176.4 (2)	C12—N1—Zn1—N4	0.6 (3)
C4—C5—N2—Zn1	−178.7 (2)	C6—N1—Zn1—N4	−174.87 (16)
C6—C5—N2—Zn1	2.4 (3)	C12—N1—Zn1—O3	169.9 (2)
C19—C18—N3—C22	−0.2 (4)	C6—N1—Zn1—O3	−5.6 (4)
C19—C18—N3—Zn1	−176.8 (2)	Cl2—O3—Zn1—O2	−42.73 (19)
C21—C22—N3—C18	0.5 (4)	Cl2—O3—Zn1—N3	120.5 (2)
C23—C22—N3—C18	−179.0 (2)	Cl2—O3—Zn1—N2	−141.1 (2)
C21—C22—N3—Zn1	177.5 (2)	Cl2—O3—Zn1—N4	40.50 (19)
C23—C22—N3—Zn1	−2.0 (3)	Cl2—O3—Zn1—N1	−129.4 (2)
C28—C27—N4—C23	−2.4 (4)		
